# Effect of Counter Electrode in Electroformation of Giant Vesicles

**DOI:** 10.3390/membranes1040345

**Published:** 2011-11-24

**Authors:** Yukihisa Okumura, Shuuhei Oana

**Affiliations:** Department of Chemistry and Material Engineering, Faculty of Engineering, Shinshu University, 4-17-1 Wakasato, Nagano 380-8553, Japan; E-Mail: soana@mc84.shinshu-u.ac.jp

**Keywords:** electroformation, electroswelling, giant vesicles, giant liposomes, lipid membrane

## Abstract

Electroformation of cell-sized lipid membrane vesicles (giant vesicles, GVs), from egg yolk phosphatidylcholine, was examined varying the shape of the counter electrode. Instead of a planar ITO (indium tin oxide) electrode commonly used, platinum wire mesh was employed as a counter electrode facing lipid deposit on a planar formation electrode. The modification did not significantly alter GV formation, and many GVs of 30–50 μm, some as large as 100 μm, formed as with the standard setup, indicating that a counter electrode does not have to be a complete plane. When the counter electrode was reduced to a set of two parallel platinum wires, GV formation deteriorated. Some GVs formed, but only in close proximity to the counter electrode. Lower electric voltage with this setup no longer yielded GVs. Instead, a large onion-like multilamellar structure was observed. The deteriorated GV formation and the formation of a multilamellar structure seemed to indicate the weakened effect of the electric field on lipid deposit due to insufficient coverage with a small counter electrode. Irregular membranous objects formed by spontaneous swelling of lipid without electric voltage gradually turned into multilamellar structure upon following application of voltage. No particular enhancement of GV formation was observed when lipid deposit on a wire formation electrode was used in combination with a large planar counter electrode.

## 1. Introduction

Large lipid membrane vesicles of a size comparable to a biological cell are known as giant vesicles (GVs) and used in various studies as a model membrane [1,2]. Various preparation methods of GVs have been reported [2], and recently, electroformation or electroswelling frequently appears as a convenient and reliable procedure to obtain many GV of larger size (typically 10–100 μm in diameter) [2,3,4,5,6,7,8,9,10,11,12,13,14,15,16,17,18,19,20,21].

The electroformation procedure is based on swelling of membrane-forming lipid deposit on an electrode assisted by applied electric voltage. Two electrode setups have been commonly used in electroformation. Historically, electroformation was first studied using two platinum wires as parallel electrodes in a water-filled trough [4,5,6]. In this setup, the top of a formation chamber may remain open, and physical access to forming GVs is readily available. To date, the wire electrode setup has been conveniently used when direct manipulation of GVs formed on electrode is necessary, such as in microinjection [16]. Another commonly used setup is a chamber that consists of two pieces of indium tin oxide (ITO) coated planar electrodes placed facing each other [7,9,10,11,12]. This planar electrode setup has the advantage of producing more vesicles at one time because of the larger surface area than a wire electrode setup. On the other hand, the structure of the setup is semi-closed, and physical access to the inside of a chamber is limited although modification of the setup to a flow chamber made replacement of the aqueous contents in a chamber possible [17,18,19].

In both setups, a pair of two similar electrodes is usually employed. Either electrode may be a formation electrode with lipid deposit, and the other works as a counter electrode. Electroformation occurs on an electrode of negative polarity [4,5,6,21]. When ac voltage is used, to obtain more GVs, lipid is often deposited on both electrodes so that an electrode alternatively functions as formation and counter electrodes.

Previously, we reported electroformation of GVs using ITO-coated PET as electrode material in a planar electrode setup [21]. In the study, a part of the counter electrode was removed to make observation windows. GV formation was still observed, and the result suggests that the size of a counter electrode could be different from that of a formation electrode.

This encouraged us to investigate how modification of a counter electrode could affect electroformation. In the present study, we examined electroswelling of lipid layer that was deposited on a planar ITO coated electrode using platinum wire mesh or wires as a counter electrode. Also, electroformation on a wire formation electrode in combination with a planar counter electrode was tested.

## 2. Results and Discussion

### 2.1. Electroswelling of Lipid Layer on a Planar Electrode with a Counter Electrode of Wire Mesh

**Figure 1:**
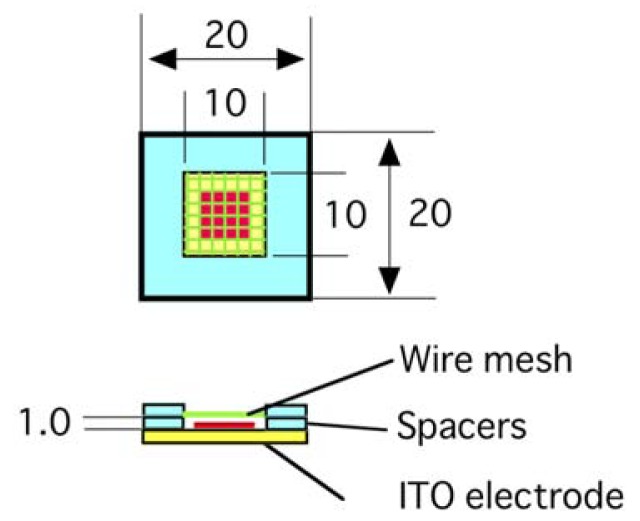
An electroformation chamber with a planar indium tin oxide (ITO) formation electrode and a counter electrode of wire mesh. Lipid (shown in red) was deposited on the formation electrode. The unit of length is millimeter.

Pt wire mesh (the diameter of the wire, 0.20 mm and the opening of the mesh 1.5 mm) was placed 1.0 mm above the surface of a planar formation electrode with lipid deposit (egg phosphatidylcholine) and used as the counter electrode (Figure 1).

**Figure 2:**
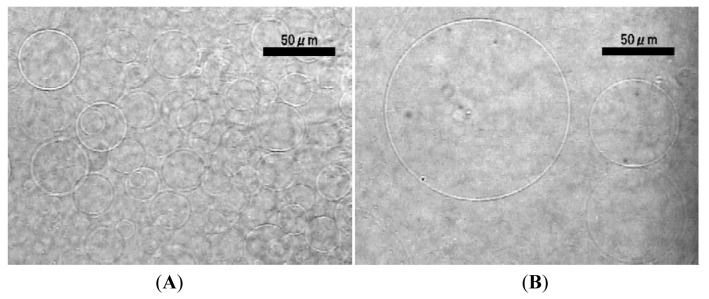
Typical GVs formed from egg phosphatidylcholine deposited on a planar ITO electrode using wire mesh as the counter electrode (**A**, 110 min) upon application of ac voltage (5.0 Vpp, 2.0 Hz). Some GVs were as large as 100 μm (**B**, 120 min).

Application of ac voltage (5.0 Vpp (peak-to-peak), 2.0 Hz) started vibration of deposited lipid layer in synchronization with the oscillation of the electric voltage and resulted in ordinary GV formation after 90–120 min (Figure 2). The typical diameter of the GV was 30–50 μm (Figure 2(A)). Some GVs as large as 100 μm were also observed (Figure 2(B)). The electroformation was comparable with that using a planar ITO-glass as a counter electrode [7]. The present result indicates that the counter electrode does not have to be a full plane, and this is consistent with the previous observation with a partial planar ITO counter electrode [21].

### 2.2. Electroswelling of Lipid Layer on a Planar Electrode with Wire Counter Electrodes

**Figure 3:**
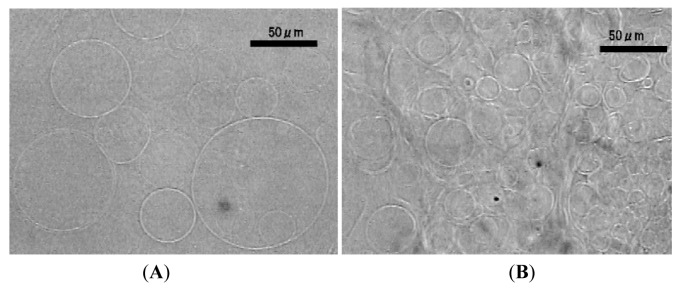
GVs formed from lipid deposit on a planar electrode directly under a double wire counter electrode (**A**, 100 min) upon application of ac voltage (5.0 Vpp, 2.0 Hz). Membranous objects of irregular shapes were found in the region distant from the counter electrode (**B**).

The counter electrode was simplified to two parallel Pt wires (diameter 0.50 mm) separated 3.0 mm and placed 1.0 mm above a formation electrode. After application of electric voltage (5.0 Vpp, 2.0 Hz), some GVs formed but only on the area directly under the double wire counter electrode (Figure 3(A)). In other region, no well-formed GVs were observed, and irregular membranous objects were mostly seen (Figure 3(B)).

The nominal surface area of the double wire counter electrode (two wires of diameter 0.50 mm and their total length 20 mm) was comparable to that of the mesh used above (fourteen wires of diameter 0.20 mm and the total length 140 mm). For efficient GV formation, it is necessary that a counter electrode should cover a broad area over lipid deposit.

**Figure 4:**
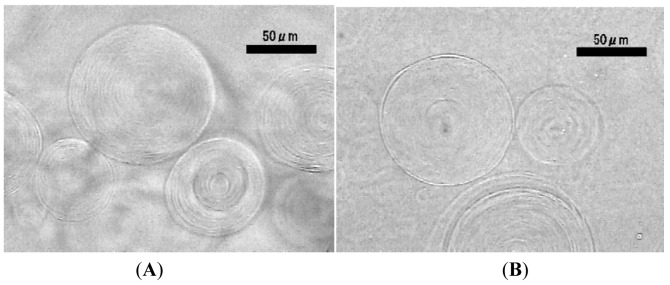
Multilamellar membranous objects formed from lipid deposit on a planar ITO formation electrode by applying ac voltage (3.0 Vpp, 2.0 Hz) using a double (**A**, 120 min) or a single (**B**, 110 min) wire counter electrode.

When the applied electric voltage was reduced from 5.0 Vpp to 3.0 Vpp, even with the closer placement of the counter electrode (0.3 mm above the surface of the formation electrode) to lipid deposit, the setup no longer yielded GVs. Instead, irregular membranous objects were observed (Figure 4(A)) under the conditions. Some of the onion-like objects were as large as 100 μm. Similar formation of multilamellar structure was also seen when the counter electrode was further simplified to a single wire (Figure 4(B)).

The effect of electric field is strongest directly under the wire counter electrode and quickly diminishes as the distance from the electrode becomes larger. Although the exact mechanism of electroformation is yet to be elucidated, electroformation seems to involve at least two steps. The first step is separation of floating lipid bilayer under the influence of applied electric field. The step has been studied both theoretically and experimentally [22,23,24]. The second step is inflation of the separated membrane to a large vesicle. The formation of multilamellar structure observed in the present study could be indication of insufficient inflation. The result suggests that the inflation process should require a stronger effect of electric field than the first step. This is understandable because the process may need transport of large mass if it is driven by electroosmotic effect as previously suggested [4,5].

**Figure 5:**
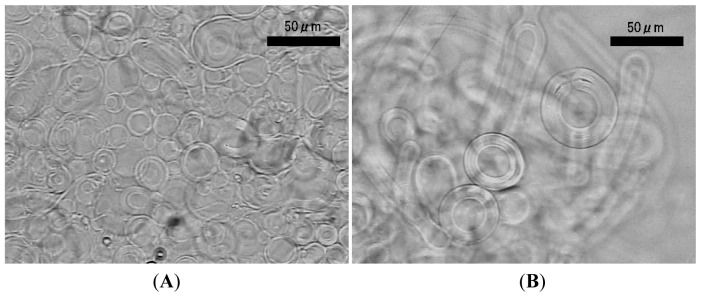
Irregular membranous objects formed by spontaneous swelling of lipid deposit on a planar formation electrode without applied electric voltage (**A**, 120 min). Curling myelin-like structures to multilamellar ones occurred upon following application of ac voltage (3.0 Vpp, 2.0 Hz) using a single wire counter electrode (**B**).

When lipid deposit on the planar formation electrode was left without electric voltage, spontaneous swelling of the lipid layer formed mostly oligolamellar objects (Figure 5(A)). Kuribayashi and coworkers also reported similar irregular membranous structure upon gentle hydration of lipid in deionized water [18]. Interestingly, applying electric voltage (3.0 Vpp, 2.0 Hz) at this point using a single wire counter electrode induced morphological change of the irregular membranous objects. Myelin-like figures that were curling to multilamellar objects were observed (Figure 5(B)). The result indicates that the electric field, even weakened by a small counter electrode, could have an effect on lipid swelling and the morphology of inflating lipid membrane.

Girard and coworkers previously showed that GVs obtained through electroformation were unilamellar [10]. Compared to a multilamellar object observed in the present study, a unilamellar GV is more suitable for a model of biological membrane. It has a single spacious inner aqueous phase and can accommodate a large amount of substances. Also, it has clear distinction between the outer and inner surfaces of membrane. This feature is essential for construction of a model system with vectorial nature such as a reconstituted trans-membrane channel. Meanwhile, multilamellar structures may be useful in other applications, for example, a carrier of membrane bound substances, making good use of their densely packed membranes inside.

### 2.3. Electroswelling of Lipid Layer on a Wire Electrode with a Planar Counter Electrode

**Figure 6:**
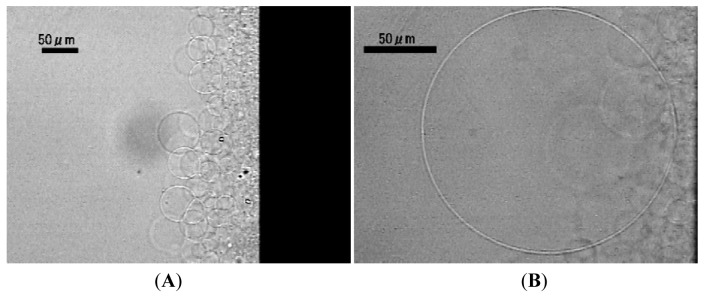
GV formation from lipid deposit on a wire formation electrode upon application of ac voltage (3.0 Vpp, 2.0 Hz) with a planar counter electrode (**A**, 100 min). A GV as large as 180 μm (**B**, 110 min) was observed. The wire electrode appears as a dark shadow on the right hand side of the image frame.

To examine a possible effect of a counter electrode that is larger than a formation electrode, using the same setup of electrodes in the experiments above, lipid was deposited on a wire formation electrode, and the planar electrode was used as a counter electrode. In this case, although the planar counter electrode has larger surface area than a wire counter electrode in a standard wire electrode setup, electroformation of GVs occurred in an ordinary manner (Figure 6). Upon application of ac voltage (3.0 Vpp, 2.0 Hz) between the electrodes, the swelling lipid layer on the wire formation electrode oscillated synchronizing with the alternation of the polarity of the applied electric field, and well-formed GVs of the diameter of 20–60 μm, which is also typical of electroformation on a wire formation electrode, were obtained. Some GV were as large as 180 μm. No particular enhancement of electroformation by the large size of the counter electrode was observed.

## 3. Experimental Section

### 3.1. Materials

Phosphatidylcholine extracted and purified from egg yolk (eggPC) was obtained from Avanti Polar Lipids (Alabaster, AL, USA). The purity of the phospholipids was checked using thin layer chromatography on silica gel plate (Silicagel 70 Plate-Wako from Wako Pure Chemicals (Osaka, Japan)) developed by a mixture of chloroform, methanol and water (65:25:4 v/v/v) as the solvent, and only a single spot was detected. Platinum wires were obtained from Nilaco (Ginza, Tokyo, Japan). ITO-coated glass was purchased from or Sigma-Aldrich (St. Louis, MI, USA). Methanol was of the analytical grade and a product of Wako Pure Chemicals.

### 3.2. Electroswelling of Lipid

For an experiment with a wire mesh counter electrode, a mesh made of platinum wire (diameter 0.20 mm, opening 1.5 mm) was placed 1.0 mm above an ITO-glass formation electrode as schematically shown in Figure 1. A methanolic solution of eggPC (5.0 mg/mL, 2.0 μL) was spread in the area of 6 mm × 6 mm on the electroconductive surface of the formation electrode. In experiments using combination of wire and planar ITO electrodes, the mesh was replaced by single or double parallel linear Pt wire (diameter 0.50 mm). In an experiment using a wire formation electrode in combination with a planar counter electrode, an eggPC solution (5.0 mg/mL, 1.0 μL) was deposited on the electrode wire (length 10 mm). Assuming uniform distribution of lipid deposit on electrode surface, the amount of lipid per area can be calculated as 0.32 μg/mm^2^ for the wire electrode and 0.28 μg/mm^2^ for a planar one.

In all the cases, after depositing lipid, the thin lipid layer was first dried spontaneously and then further under the reduced pressure of a water aspirator. The chamber was then filled with Milli-Q grade ultrapure water, and sinusoidal ac voltage (3.0 or 5.0 Vpp (peak-to-peak), 2.0 Hz unless otherwise noted) was applied between formation and counter electrodes from a function generator (Kenwood TMI FG-272, Yokohama, Japan). Lipid swelling was observed with an inverted optical microscope (Olympus IX-50, Tokyo, Japan) equipped with phase contrast and digital image enhancement options.

## 4. Conclusions

The present study revealed that in electroformation of GVs on a planar formation electrode, the counter electrode did not have to be a complete plane. A mesh of thin wire, if it covers sufficient area over lipid deposit on a formation electrode, should be sufficient as a counter electrode. Simplifying a counter electrode to a double parallel or a single wire significantly deteriorated GV formation. Under these conditions, a membranous multilamellar structure formed, suggesting insufficient effect of the electric field that was weakened by the small counter electrode on the separation and/or inflation of lipid membrane. Although inadequate for electroformation of GVs, the weakened electric field could still affect electroswelling of lipid. Application of electric voltage with the wire counter electrode to lipid that had first swelled spontaneously without voltage resulted in morphological change, curling myelin-like objects to multilamellar ones. Electroformation on a wire formation electrode used in combination with a large planar counter electrode occurred in an ordinary manner, and there seemed to be no particular enhancement of electroformation by using a large counter electrode.
